# Comparative metabolomic analysis in roots of natural and cultivated *Astragalus membranaceus* in Mongolia

**DOI:** 10.1186/s40529-026-00490-6

**Published:** 2026-01-26

**Authors:** Munkhtsetseg Tsednee, Chao-Yu Hsu, Shingo Sakamoto, Nobutaka Mitsuda, Bolor Tsolmon, Ming-Tsair Chan, Chuan-Ming Yeh

**Affiliations:** 1 Institute of Chemistry and Chemical Technology, Mongolian Academy of Sciences, Ulaanbaatar, 13330 Mongolia; 2 Division of Urology, Department of Surgery, Tungs’ Taichung MetroHarbor Hospital, Taichung, 435403 Taiwan; 3 Biomanufacturing and Process Research Center, National Institute of Advanced Industrial Science and Technology (AIST), Tsukuba, Ibaraki 305-8566 Japan; 4 Academia Sinica Biotechnology Center in Southern Taiwan, Agricultural Biotechnology Research Center, Academia Sinica, Tainan, 711010 Taiwan; 5 Institute of Molecular Biology, National Chung Hsing University, Taichung, 402202 Taiwan; 6 Advanced Plant and Food Crop Biotechnology Center, National Chung Hsing University, Taichung, 402202 Taiwan; 7 Research Center for Environmental Changes, Academia Sinica, Taipei, 115201 Taiwan

**Keywords:** *Astragalus*, Cultivation, GC–MS, Metabolomics, Polysaccharide

## Abstract

**Background:**

*Astragalus membranaceus*, a valuable medicinal plant, is widely used in the pharmaceutical, food, and nutritional industries due to its rich bioactive compounds. Its increasing demand has led to extensive cultivation of *A. membranaceus* to supplement natural resources and ensure a stable supply. However, comparing the metabolic characteristics of natural and cultivated plants is essential for understanding their quality, authenticity, and potential pharmacological differences.

**Results:**

We conducted a comparative analysis of polysaccharide and monosaccharide composition and untargeted metabolite profiling in the roots of natural and cultivated *A. membranaceus* plants in Mongolia. The levels of alcohol soluble total polysaccharides and the major abundant monosaccharides were similar between natural and cultivated *A. membranaceus* roots. Whereas several less abundant monosaccharides showed reduced levels in the cultivated roots. Untargeted metabolomic profiling identified a total of 157 metabolites, among which 42 and 35 were differentially accumulated in natural and cultivated roots, respectively. Most metabolites showed increased levels in the cultivated roots; however, 32 metabolites were enriched in natural roots. Functional pathway enrichment revealed distinct metabolic features between the two root types. In natural roots, pathways related to stress response, biosynthesis of secondary metabolites, and energy production were enriched. In cultivated roots, the enriched metabolic pathways were linked to primary metabolism, growth, and energy production.

**Conclusions:**

Our findings reveal distinct metabolic characteristics between natural and cultivated *A. membranaceus* roots, likely shaped by differences in growth environments, soil conditions, and adaptive metabolic reprogramming. These results provide a valuable reference for evaluating, authenticating, and distinguishing natural and cultivated *A. membranaceus* roots, and offer insights into their pharmacological potential and quality control.

**Supplementary Information:**

The online version contains supplementary material available at 10.1186/s40529-026-00490-6.

## Introduction

*Astragalus membranaceus* (Fisch.) Bunge., a perennial herb belonging to the Fabaceae family, is widely distributed in temperate and arid regions of Asia, Europe, and North America (Sheik et al. 2021). It has long been used in traditional medicine across Asian countries with different names, e.g., as “huangqi” in China, “хунчир-hunchir” in Mongolia, “ogi” in Japan, and in Korea, for various therapeutic purposes, including treating weakness, wounds, cold, fever, chronic fatigue, anemia, and detoxification of the body (Su et al. 2021). Pharmacological studies have further confirmed that *A. membranaceus* tissues exhibit diverse biological activities, such as antiviral (Zhu et al. 2009), antioxidant (Yan et al. 2010), anti-inflammatory (Lai et al. 2012), antidiabetic (Lu et al. 2015), anti-aging (Liu et al. 2017), and immunomodulatory activities (Cho and Leung 2007; Li et al. 2022b). In addition, *A. membranaceus* has demonstrated cardiovascular protective effects (Su et al. 2021), neuroprotective effects (Abd Elkader et al. 2022), alleviation of Parkinson’s disease (Yang et al. 2019), and anti-cancer activities against stomach, liver, lung, breast, colorectal, ovarian, cervical, and lymphoma cancers (Li et al., 2020a; Sheik et al. 2021; Zhang et al. 2020). *A. membranaceus* is, therefore, known as one of the most valuable and widely utilized medicinal herbs worldwide.

The major bioactive phytoconstituents in *A. membranaceus* include polysaccharides, saponins, phenolics/flavonoids, and triterpenes (Salehi et al. 2021; Su et al. 2021). Its roots are particularly rich in polysaccharides, most of which are heteropolysaccharides composed of rhamnose, xylose, ribose, galactose, glucose, and mannose (Sheik et al. 2021; Tian et al. 2025). These polysaccharides have been widely reported to exhibit immunomodulatory and antidiabetic activities (Chang et al. 2020; Tian et al. 2025). In addition, the triterpenoid saponins, such as astragaloside I, II, III, and IV, have been identified in *A. membranaceus* roots and are known for their cardioprotective effects, together with other flavonoids including calycosin, ononin, and calycosin 7-*O*-beta-D-glucopyranoside (Li et al. 2017; Wang et al. 2020). Several isoflavones such as calycosin, ononin, campanulin, and formononetin in *A. membranaceus* plants have also been reported to possess anti-tumor and anticancer activities (Li et al. 2020b; Zhu et al. 2009).

In recent decades, the use of *A. membranaceus* roots has expanded significantly in the food, nutrition (Ny et al. 2021; Shahrajabian et al. 2019), and cosmetic industries (Kim et al. 2011; Tsao et al. 2017). Consequently, increasing demand for raw materials, particularly the root tissues, has placed wild *A. membranaceus* populations at risk of becoming endangered in several countries (Dong et al. 2024; Kim et al. 2014; Qin et al. 2020). In Mongolia, *Astragalus* species are officially listed as endangered (Baasanmunkh et al. 2021). To protect natural populations, cultivation of *A. membranaceus* was initiated several years ago in Khentii province, located in eastern Mongolia (Odontuya et al. 2020). More recently, the utilization of cultivated *A. membranaceus* roots has increased markedly, particularly for food and nutrition supplements.

Environmental factors, including various abiotic and biotic stressors, are known to strongly influence the composition and abundance of phytocompounds in plants (Yang et al. 2020). Therefore, it is essential to examine and evaluate the overall metabolite constituents, as well as the main bioactive compounds, in both natural and cultivated plants to ensure their quality at the metabolic level, as reported in similar studies conducted in China (Bi et al. 2020; Li et al. 2024). However, to date, no comparative metabolomic analysis has been reported on the natural and cultivated *A. membranaceus* plants in Mongolia. Here, we present the first comparative study on the polysaccharide and monosaccharide analysis and untargeted metabolite profiling of root samples collected from natural and cultivated *A. membranaceus* in Mongolia.

## Materials and methods

### Plant materials

Natural *A. membranaceus* plants were collected from Zavkhan province, Mongolia, during their active growth season in July 2021. The root tissues were air-dried immediately, while avoiding direct sunlight, and used for the study. The chopped and dried raw root tissues of cultivated *A. membranaceus*, without any preprocessing, were purchased from commercial stores in Ulaanbaatar city, Mongolia. The cultivated *A. membranaceus* were grown in the cultivation field of Kherlenbayan soum in Khentii province, and the cultivation was maintained stably over eight years.

### Chemicals and reagents

All organic solvents and chemicals (with analytical grades), including methanol, ethanol, chloroform, benzene, N, O-Bis(trimethylsilyl)-trifluoroacetamide, trimethylchlorosilane, ribitol, and galacturonic acid were purchased from Sigma-Aldrich (Chemie GmbH at Munich, Germany and Sigma Aldrich at St Louis, MO, USA). Monosaccharide standards were purchased from Kanto Chemical Inc. (Japan).

### Determination of alcohol-soluble polysaccharide (ASP) content

For the quantification of ASPs, 500 g of homogenized root samples were treated sequentially with benzene: methanol, C_6_H_6_:CH_3_OH (2:1, v/v), and 80% ethanol, at room temperature using a Soxhlet apparatus for 4–5 h in each extraction step. After removing the extractable substances, the remaining root residues were continuously extracted with distilled water at 80°C for 3–4 h and further filtered to obtain clean extracts. The aqueous extract was then concentrated to one-fifth of the volume using a vacuum evaporator and then precipitated with 95% ethanol (extract: ethanol ratio of 1:5). The ASP precipitates were washed with 70% ethanol 3–4 times, dialyzed with distilled water for sufficient hours using a membrane dialyzer, and dried using a freeze-dryer. The analysis was conducted using *n* = 3 biological replicates from both natural and cultivated root samples. The total ASP content, presented by yield (%), was calculated using the dried ASP precipitates and the calculation formula below.

$${\rm{ASP}}\>{\rm{content}}\>(\% ){\rm{ = }}\left( {{{{\rm{Weight}}\>{\rm{of}}\>{\rm{dried}}\>{\rm{ASP}}} \over {{\rm{Initial}}\>{\rm{sample}}\>{\rm{weight}}}}} \right) \times {\rm{100}}$$

The ASP contents in natural and cultivated *A. membranaceus* roots were further presented by normalizing the values to that of natural *A. membranaceus* roots as 1.0.

### Monosaccharide analysis using Ultra-Performance Liquid Chromatography (UPLC)

The sample preparation steps, further identifications, and quantifications of monosaccharides in root samples by the UPLC approach followed a previous method (Sakamoto et al. 2015). The analysis used three biological replicates of root samples together with all the monosaccharide standards, and the analytical conditions on the UPLC instrument were also followed and performed, as described by Sakamoto et al. 2015; at the Bioproduction Research Institute, AIST, Japan.

### Metabolomic profiling using Gas Chromatography–Mass Spectrometry (GC–MS)

For the GC–MS analysis, total metabolites from 200 mg of homogenized root tissues were extracted with 80% methanol at room temperature under sonication for 30 min. The sample extracts were then collected by centrifugation at 13,000 rpm for 15 min and freeze-dried completely after adding the internal standard, Ribitol, together with the extraction buffer (blank control) samples. Further derivation steps of dried extracts (using methoxyamine, N, O-Bis(trimethylsilyl)trifluoroacetamide with 1% trimethylchlorosilane) and analytical conditions on the GC–MS instrument were followed by our previous method conditions (Erdenetsogt et al. 2024). As natural plants are difficult to obtain, three replicates of root samples, collected from natural and cultivated *A. membranaceus* plants, were prepared, each replicate consisting of chopped and pooled roots from 3 to 5 individual plants. Quality control analysis used pooled sample mixtures and blank control samples.

### Metabolomic data analysis

Principal component analysis (PCA), cluster heatmap analysis, metabolic pathway enrichment analysis, z-score, and *t*-test were performed on the relevant metabolic data using statistical analysis software such as SigmaPlot 12.0, OriginPro, and the web-based software MetaboAnalyst 6.0. The log2-transformed metabolite data were used for the data analysis. The heat-map was generated without False Discovery Rate (FDR) correction to directly visualize the abundance patterns. For pathway enrichment analyses, FDR-corrected (FDR < 0.05) *p-*values were applied to each metabolite to reduce false positives.

## Results

### Polysaccharide and monosaccharide analysis in roots of natural and cultivated *A. membranaceus*

**Fig. 1:**
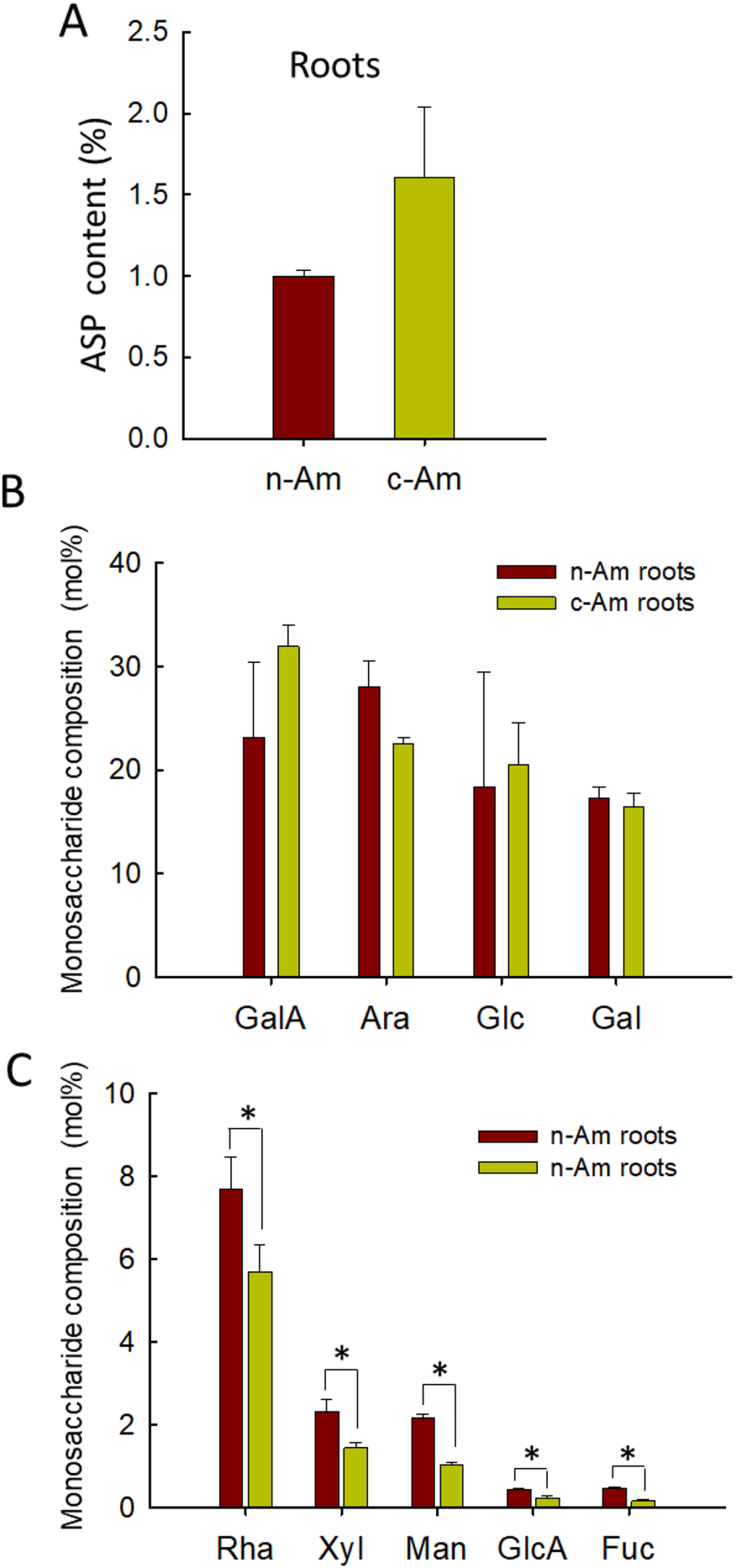
Saccharide analysis in natural and cultivated *A. membranaceus* roots. (**A**) The contents of alcohol-soluble polysaccharides (APSs) in natural (n-Am) and cultivated (c-Am) *A. membranaceus* roots are normalized to that of n-Am roots as 1.0. (**B**) and (**C**) Major abundant and less abundant polysaccharides, respectively, were determined in n-Am and c-Am roots. Data are the mean of *n* = 3 replicates. Asterisks indicate significant changes in the compared samples (Student’s *t*-test; *, *P* < 0.05). GalA = Galacturonic acid; Ara = Arabinose; Glc = Glucose; Gal = Galactose; Rha = Rhamnose; Xyl = Xylose; Man = Mannose; GlcA = Glucuronic acid; Fuc = Fucose

Since the identified bioactive phytocompounds in *A. membranaceus* are accumulated predominantly in their root tissues (Salehi et al. 2021; Tian et al. 2025), all analyses in this study were conducted using root samples collected from the natural (n-Am) and cultivated (c-Am) plants. We first determined the polysaccharide contents, as they represent the major metabolites in *A. membranaceus* roots. The total contents of alcohol-soluble polysaccharides (ASPs) were determined from aqueous extracts after precipitating with ethanol. Although the total ASP content was about 1.5-fold higher in c-Am roots compared with n-Am roots, the difference was not statistically significant between the two root types (Fig. 1A). The results thus indicate that the overall ASP levels are comparable between natural and cultivated *A. membranaceus* roots.

Using UPLC–MS analysis, we further quantified the monosaccharides in the root samples. In both n-Am and c-Am roots, the predominant monosaccharides were galacturonic acid (GalA), arabinose (Ara), glucose (Glc), and galactose (Gal). Additionally, five other monosaccharides, including rhamnose (Rha), xylose (Xyl), mannose (Man), glucuronic acid (GlcA), and fucose (Fuc), were detected with relatively lower abundances. Notably, the contents of four major monosaccharides did not differ significantly between n-Am and c-Am roots (Fig. 1B). However, the less abundant monosaccharides showed significant reductions in c-Am roots compared with n-Am roots (Fig. 1C). Since the major monosaccharides remained at similar levels between natural and cultivated roots, the decreases in less abundant monosaccharides in the c-Am roots are likely to have a limited impact on the overall polysaccharide contents and quality. Of note, 4-O-methyl-D-glucuronic acid (mGlcA), another monosaccharide, was not detected in either n-Am or c-Am roots.

### Metabolomic profiling of natural and cultivated *A. membranaceus* roots

**Fig. 2:**
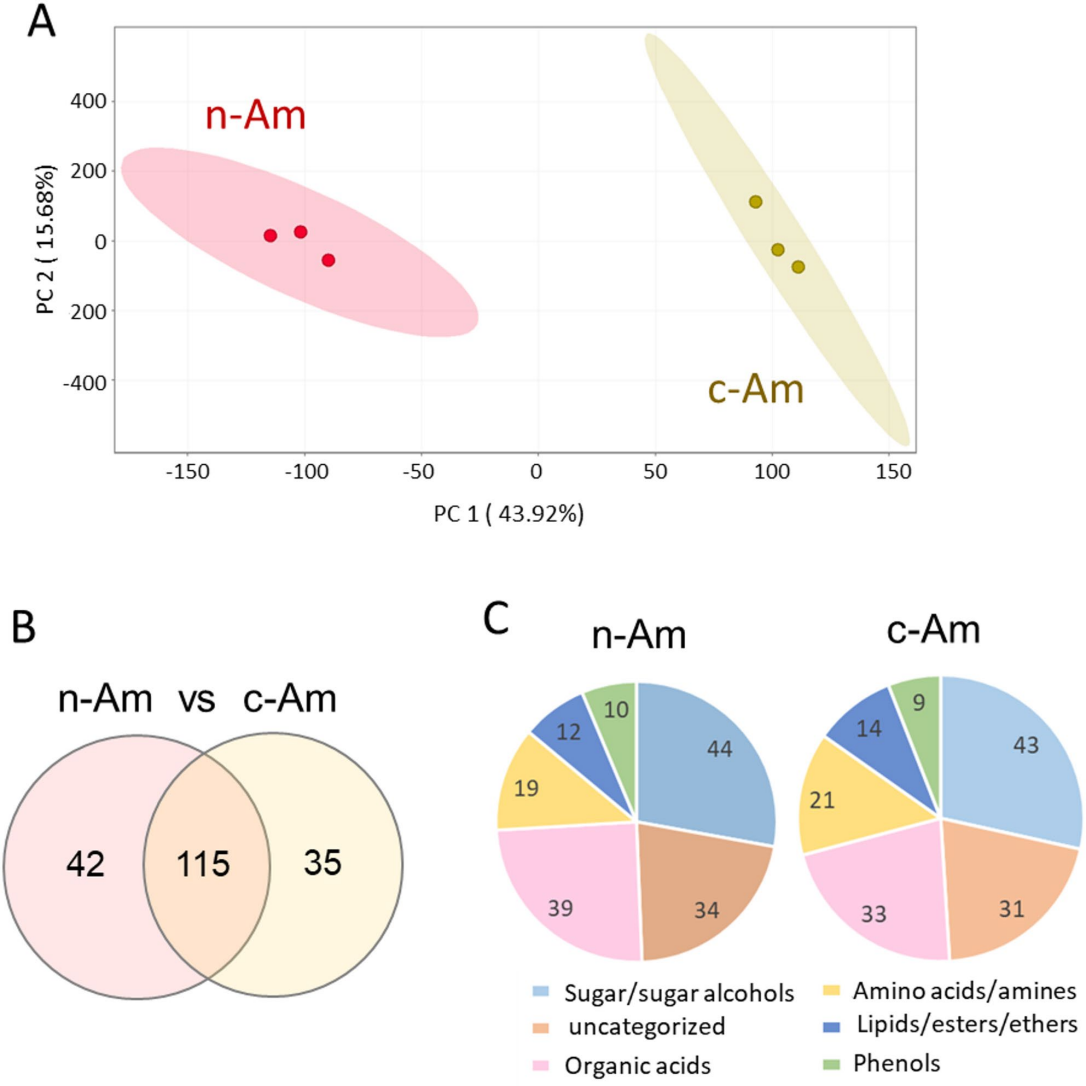
Untargeted metabolite profiling in root extracts of natural and cultivated *A. membranaceus*. (**A**) Principal component analysis of root metabolites of natural (n-Am) and cultivated (c-Am) *A. membranaceus.* (**B**) Venn diagram of differentially accumulated metabolites in n-Am and c-Am roots. (**C**) Types and numbers of metabolites identified in n-Am and c-Am roots

To further compare the overall metabolite features in n-Am and c-Am roots, we next performed a non-targeted GC–MS metabolite profiling analysis using 80% methanol extracts of root samples. Principal component analysis (PCA) revealed a clear separation between n-Am and c-Am roots, indicating significant differences in their metabolite compositions (Fig. 2A). In total, GC–MS profiling identified 157 annotated metabolites in n-Am roots and 150 in c-Am roots. Of them, 42 and 35 metabolites were differentially identified in n-Am and c-Am roots, respectively (Fig. 2B). The identified metabolites were then classified into six categories, including sugar/sugar alcohols (44 in n-Am, 43 in c-Am), organic acids (39 in n-Am, 33 in c-Am), amino acids/amines (19 in n-Am, 21 in c-Am), lipids/esters/ethers (12 in n-Am, 14 in c-Am), phenols (10 in n-Am, 9 in c-Am), and uncategorized (34 in n-Am, 31 in c-Am) (Fig. 2C). The list of differentially identified metabolites is provided in the Supplementary Tables 1, and is discussed in detail in the following sections. Together, our non-targeted metabolomic profiling revealed that at the metabolite level, although n-Am and c-Am roots are overlapped largely, they still carry distinct sets of differentially accumulated specific metabolites in their roots.

### Sugars and sugar alcohols in n-Am and c-Am roots

**Fig. 3:**
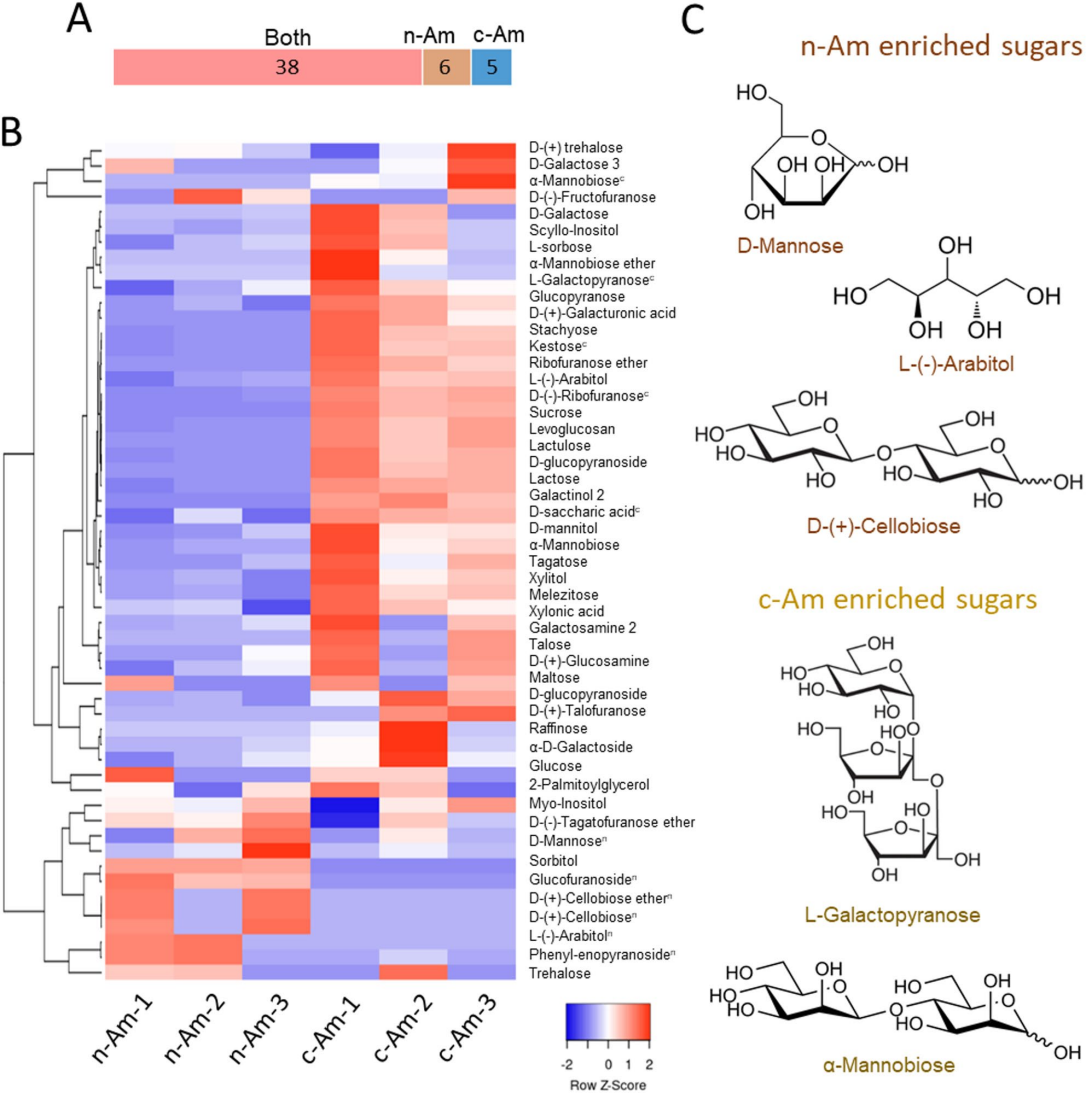
Accumulation of sugars and sugar alcohols in natural and cultivated *A. membranaceus* roots. (**A**) Bar chart of the number of sugars and sugar alcohols between natural (n-Am) and cultivated (c-Am) roots. (**B**) Clustering heat map of identified sugars and sugar alcohols in n-Am and c-Am roots. Three biological replicates were included in the experiment. The color bar represents Z scores of metabolite levels. On the upper right of the metabolites, the superscript letters “n” and “c” denote those metabolites detected only in n-Am and c-Am roots, respectively. (**C**) Specifically enriched sugar and sugar alcohols in n-Am and c-Am roots

Furthermore, using our GC–MS metabolomic profiling data, we examined the relative abundances of the identified metabolites in n-Am and c-Am roots. For this purpose, we employed a heat-map analysis to illustrate the differential accumulation of metabolites in the two types of roots. In n-Am roots, we identified 44 sugars and sugar alcohols, and 6 of them showed specific accumulations in their roots only (Fig. 3A). We also detected differential accumulation patterns of 5 sugars and sugar alcohols out of 43 identified in c-Am roots. The heat-map analysis data showed that the accumulation patterns of these sugars and sugar alcohols are different between natural versus cultivated roots (Fig. 3B). Interestingly, the majority of them (more than 2/3 of the identified sugar and sugar alcohols) showed increases in c-Am roots compared to those in n-Am roots (Fig. 3B). This suggests that the accumulation of sugars and sugar alcohols in c-Am roots has increased after its cultivation from wild habitats to the field. Conversely, 10 sugars and sugar alcohols were found to be more abundant in n-Am roots than in c-Am roots.

Consistently, the relative abundances of six differentially accumulated sugars (D-mannose, glucofuranoside, D-(+)-cellobiose ether, D-(+)-cellobiose, L-(-)-arabitol, and phenyl-eopyranoside) in n-Am roots, and five sugar alcohols (α-mannobiose, L-galactopyranose, kestose, D-(-)-ribofuranose, and D-saccharic acid) in c-Am roots also showed distinct accumulation patterns in the heat map analysis (Fig. 3B). In plant roots, the accumulation of specific sugars and sugar alcohols could result from various factors, such as growth regulation, sugar signaling, nutrient response, and rhizosphere interactions (Keunen et al. 2013). Therefore, the observed differential accumulations in the two root types suggest possible alterations in sugar metabolism and transport regulation between n-Am and c-Am roots. Moreover, these metabolites, as shown with representatives in n-Am (D-mannose, L-(-)-arabitol, and D-(+)-cellobiose) and c-Am roots (L-galactopyranose and α-mannobiose, Fig. 3C), may also serve as markers to distinguish natural from cultivated *A. membranaceus* roots.

### Organic acids and amino acids in roots of n-Am and c-Am

**Fig. 4:**
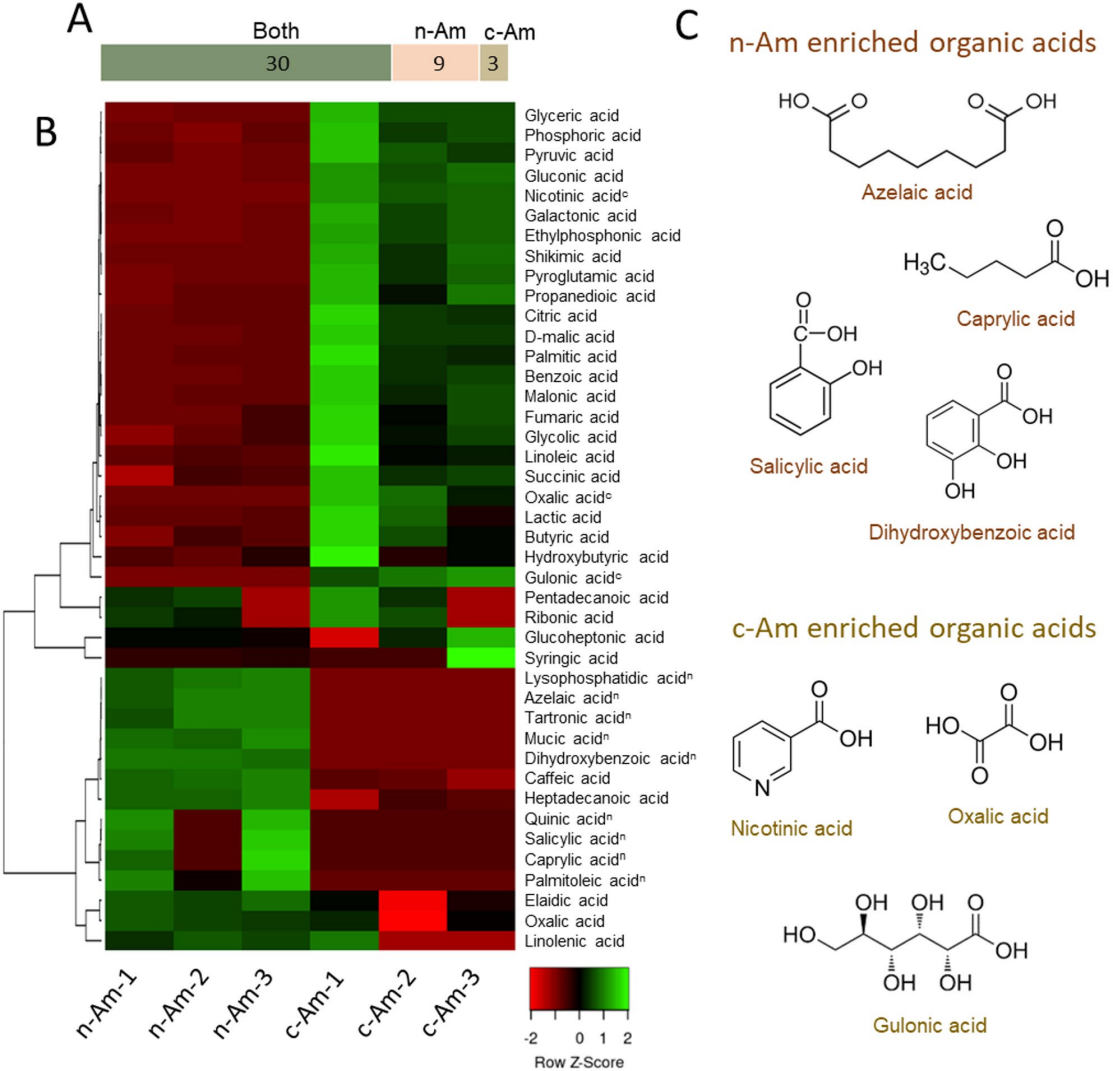
Accumulation of organic acids in natural and cultivated *A. membranaceus* roots. (**A**) Bar chart of the number of organic acids between natural (n-Am) and cultivated (c-Am) roots. (**B**) Clustering heat map of organic acids in n-Am and c-Am roots. The experiment included three biological replicates. The color bar represents Z scores for acid levels. On the upper right of the organic acids, the superscript letters “n” and “c” denote those metabolites detected only in n-Am and c-Am roots, respectively. (**C**) Specifically enriched organic acids in n-Am and c-Am roots

Using the metabolomic data, we further compared the relative abundances of organic acids, amino acids, and amines in the natural and cultivated *A. membranaceus* roots. For organic acid analysis, we combined all types of identified organic acids, including low-molecular-weight aliphatic and high-molecular-weight aromatic organic acids. A total of 39 and 33 organic acids were identified in n-Am and c-Am roots, respectively (Fig. 2C). Among them, 9 organic acids were enriched in n-Am roots, whereas 3 organic acids showed differential accumulations in c-Am roots (Fig. 4A). Heat-map analysis further revealed distinct accumulation patterns between the two root types (Fig. 4B). The majority of the organic acids (27 organic acids) exhibited higher accumulation levels in c-Am roots, whereas 15 organic acids were predominantly enriched in n-Am roots.

Intriguingly, among the organic acids enriched in n-Am roots, 9 of them (lysophosphatidic acid, azelic acid, tartonic acid, mucic acid, dihydroxybenzoic acid, salicylic acid, quinic acid, caprylic acid, and palmitoleic acid) were specifically detected and accumulated in n-Am roots. Several of these (Fig. 4C), including salicylic acid, dihydroxybenzoic acid (Huang et al., 2018), and azelaic acid (Haghpanah et al. 2024), are well known for their roles in plant immunity and defense responses against pathogens. In contrast, three organic acids (nicotinic acid, oxalic acid, and gulonic acid, Fig. 4C) were accumulated uniquely in c-Am roots. These results indicate that the metabolism of organic acids differs between natural and cultivated roots, reflecting adaptations to their varied environmental conditions.

**Fig. 5:**
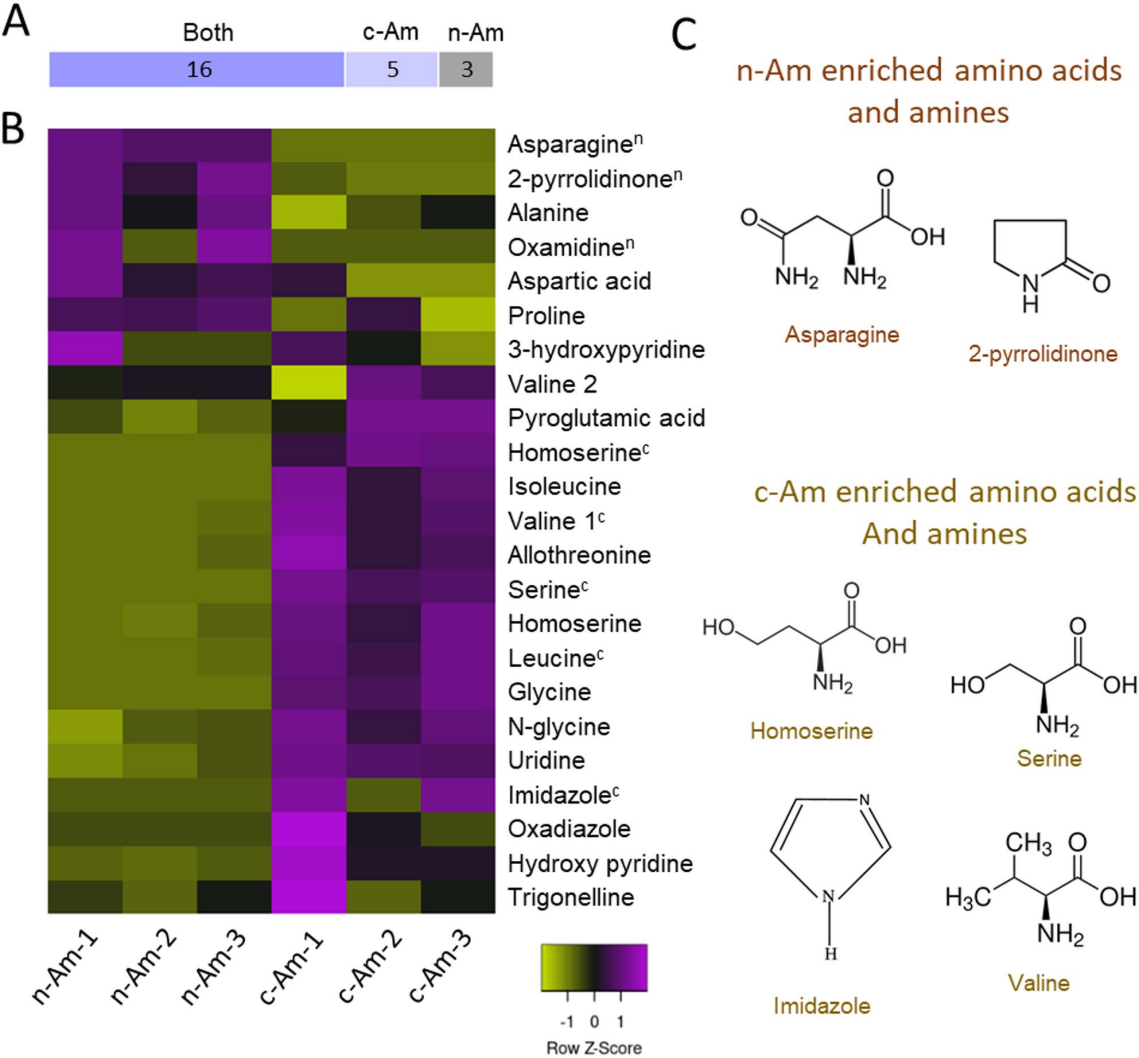
Accumulation of amino acids and amines in natural and cultivated *A. membranaceus* roots. (**A**) Bar chart of the number of amino acids in natural (n-Am), cultivated (c-Am), or both plant roots. (**B**) Clustering heat map of amino acids in n-Am and c-Am roots. The experiment included three biological replicates, and the color bar represents Z scores for levels of the amino acids and amines in the root samples. On the upper right of the amino acids, the superscript letters “n” and “c” denote those metabolites detected only in n-Am and c-Am roots, respectively. (**C**) Specifically enriched amino acids and amines in n-Am and c-Am roots

Furthermore, a total of 19 and 21 amino acids and amines were identified in n-Am and c-Am roots, respectively, and also showed distinct accumulation patterns in the heatmap analysis (Fig. 5). Among them, 3 amino acids and amines were specifically enriched in n-Am roots, whereas 5 were differentially accumulated in c-Am roots (Fig. 5A and C). Similar to the trend observed for organic acids, most of the amino acids and amines exhibited higher abundances in c-Am roots compared with n-Am roots (Fig. 5B).

### Lipids, esters, ethers, and phenolic acids in n-Am and c-Am roots

**Fig. 6:**
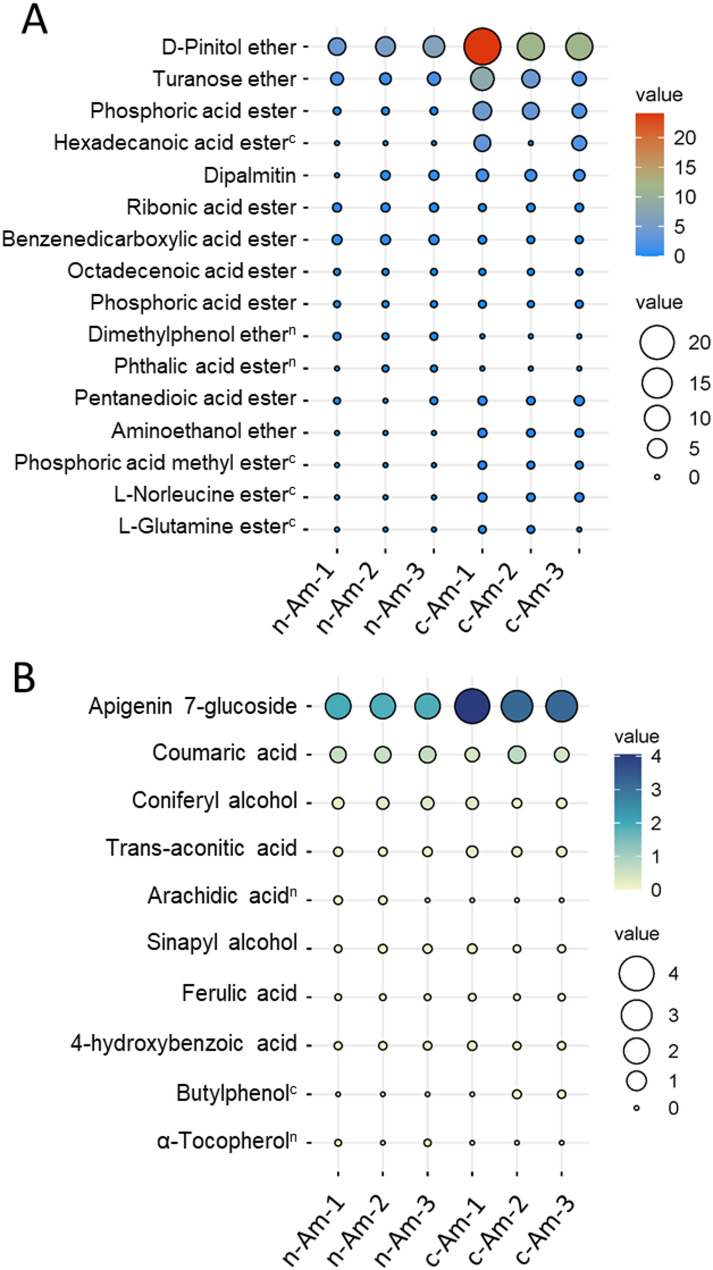
Accumulation of lipids, esters, ethers, and phenolic acids in natural and cultivated *A. membranaceus* roots. (**A**) and (**B**) Accumulations of identified lipids, ethers, and phenols, respectively, in natural (n-Am) and cultivated (c-Am) roots. Three biological replicates were included in the experiment. The colors and sizes of the balloons represent metabolite levels. On the upper right of metabolites, the superscript letters “n” and “c” denote those metabolites detected only in n-Am and c-Am roots, respectively

From our non-targeted metabolomic profiling data, we identified a relatively small number of lipids, fatty acids, esters, and ethers, totaling 12 in n-Am roots and 14 in c-Am roots (Fig. 2C). Among these, three ethers (D-pinitol ether, turonase ether, and aminoethanol ether) showed higher accumulations in c-Am roots compared with n-Am roots (Fig. 6A). Additionally, four acid esters (hexadecanoic acid ester, phosphoric acid methyl ester, L-norleucine ester, and L-glutamine ester) were accumulated specifically in c-Am roots. However, n-Am roots contained one specifically enriched ether (dimethylphenol ether) and an acid ester (phthalic acid ester).

In plants, acid esters play diverse roles in growth and development, defense response, and stress tolerance (Uranga et al. 2016; Zhou et al. 2017). Our follow-up metabolic pathway analysis may help elucidate the functional roles of these metabolites, differentiating n-Am versus c-Am roots.

Moreover, GC–MS profiling identified a total of 10 phenols and phenolic acids across both root types (Fig. 6B). Among them, arachidic acid and a-tocopherol were specifically enriched in n-Am roots, while butylphenol was detected in c-Am roots only. Although the detailed analyses of phenolic compounds would benefit from liquid chromatography coupled with MS (LC–MS), our current results suggest possible alterations in phenolic compounds between n-Am and c-Am roots.

### Metabolic pathway analysis in n-Am and c-Am roots

Finally, to gain deeper insights into the functional differences underlying the metabolic variations between natural and cultivated *A. membranaceus* roots, we performed metabolic pathway analysis using two sets of metabolites: (i) those enriched in n-Am and c-Am roots and (ii) differentially accumulated metabolites between the two root types. The annotated metabolites were analyzed using the web-based software MetaboAnalyst 6.0, which identified the predominant metabolic pathways contributing to the observed metabolic variations.

**Fig. 7:**
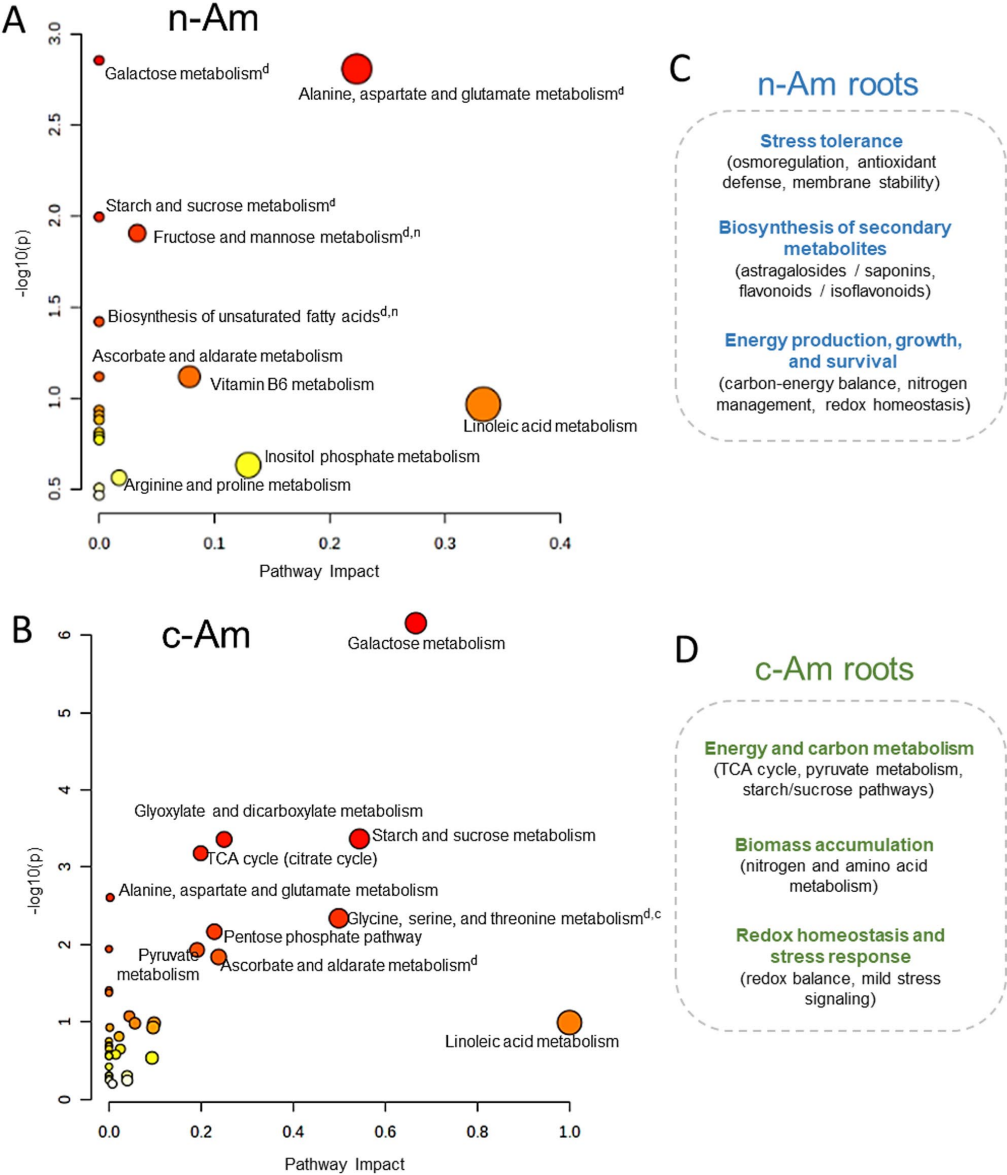
Metabolic pathway analysis in natural and cultivated *A. membranaceus* roots. (**A**) and (**B**) Metabolic pathway analysis plot created in natural (n-Am) and cultivated *A. membranaceus* (c-Am) roots using MetaboAnalyst 6.0. The pathway impact value on the *x*-axis was obtained from the pathway topological analysis. The –log10 of *p*-value on the *y*-axis was obtained from pathway enrichment analysis. The larger circles represent more significant pathway enrichments, and the darker colors represent the greater significance. On the upper right of metabolic pathways, the superscript letters “n” and “c” denote those metabolites detected only in n-Am and c-Am roots, respectively, and “d” denotes pathways identified that overlap and are associated which are overlapped but differentially accumulated metabolites. Functional roles associated with metabolic pathways identified in n-Am (**C**) and c-Am (**D**) roots

First, using 33 high-abundance metabolites enriched in n-Am roots and 95 in c-Am roots, we examined the associated metabolic pathways. In n-Am roots, the most enriched pathways included alanine, aspartate, and glutamate metabolism; galactose metabolism; starch and sucrose metabolism; fructose and mannose metabolism; biosynthesis of unsaturated fatty acids; ascorbate and aldarate metabolism; vitamin B6 metabolism; linoleic acid metabolism; inositol phosphate metabolism; and arginine and proline metabolism (Fig. 7A, Supplementary Table 2). Functionally, these pathways are linked to increased stress tolerance (supported by osmoregulation, antioxidant defense, and membrane stability), biosynthesis of secondary metabolites (including astragalosides/saponins and flavonoids/isoflavonoids), and support for energy, growth, and survival (facilitated by carbon-energy balance, nitrogen management, and redox homeostasis) (Fig. 7C). These results indicate that n-Am roots exhibit a strong metabolic orientation toward stress adaptation and energy production, while enhancing secondary metabolite biosynthesis.

In contrast, the most enriched pathways in c-Am roots were galactose metabolism; glyoxylate and dicarboxylate metabolism; citrate cycle (TCA cycle); starch and sucrose metabolism; alanine, aspartate, and glutamate metabolism; glycine, serine, and threonine metabolism; pentose phosphate pathway; pyruvate metabolism; ascorbate and aldarate metabolism; and linoleic acid metabolism (Fig. 7B, Supplementary Table 2). These pathways are associated with energy and carbon metabolism, biomass accumulation, and maintenance of stress response and redox balance (Fig. 7D). Thus, compared with n-Am roots, c-Am roots appear to emphasize metabolic regulation toward central carbon metabolism and energy production.

Next, we analyzed the pathways associated with specifically accumulated metabolites, including 41 in n-Am and 36 in c-Am roots. Notably, the identified pathways in both root types (Supplementary Table 3) overlapped with the enriched pathways shown in Fig. 7A and B (marked with the letter “d” for differential). Additionally, two pathways: fructose and mannose metabolism, and biosynthesis of unsaturated fatty acids, were detected specifically in n-Am roots, while glycine, serine, and threonine metabolism was found only in c-Am roots (marked with letters “n” and “c”, respectively, in Fig. 7A and B). This indicates that specific pathways operate differently between the two root types. Taken together, the metabolic pathway analysis revealed distinct alterations in biochemical pathways and networks between n-Am and c-Am roots.

## Discussion

The compositions and accumulations of metabolites in plants are generally shaped by their interactions with the surrounding environment and adaptation to various abiotic and biotic factors (Kessler and Kalske 2018). In *Astragalus* species, variations in bioactive metabolite contents have been previously reported across different cultivation regions in China (Bi et al. 2020; Liu and Yan 2023; Sun et al. 2020). In recent years, cultivation of *A. membranaceus* has also been introduced in Mongolia, and its roots have increasingly entered the medicinal and food markets in the country.

However, current studies on Mongolian cultivated *A. membranaceus* remain very limited, focusing primarily on the quantification of total saponin contents (Odontuya et al. 2020). Therefore, a comprehensive comparative analysis of major metabolites and overall metabolic profiles between natural and cultivated *A. membranaceus* plants in Mongolia is critically needed. Such an investigation is essential for evaluating the quality of cultivated plants, understanding the impact of environmental and cultivation conditions on metabolic composition, and identifying potential metabolic markers that can distinguish between wild and cultivated plants.

### Similar polysaccharide and monosaccharide levels in n-Am vs. c-Am roots

Our comparative analysis of polysaccharide and monosaccharide contents between natural and cultivated *A. membranaceus* roots revealed no significant changes in the total contents of alcohol-soluble polysaccharides. Similarly, the major abundant monosaccharides remained unchanged between the two root types. However, we observed a significant decrease in several less abundant monosaccharides in cultivated *A. membranaceus* roots compared to those of natural roots. To obtain more detailed insights into these variations, further investigations are needed using both types of root samples collected across different seasons and at multiple developmental stages, which will be the focus of our future studies. Moreover, since the present study was limited to root tissues, the use of different tissues, such as stems, leaves, and flowers, from both natural and cultivated plants, will provide a more complete and systematic comparative analysis at the overall plant level.

### Distinct metabolic features in c-Am and n-Am roots

Furthermore, our untargeted metabolomics profiling analysis revealed that the natural and cultivated *A. membranaceus* roots exhibited significant metabolic differences. The identified metabolic compounds, including sugars/sugar alcohols, organic acids, amino acids, phenolic acids, and acid esters, showed distinct accumulation patterns between n-Am and c-Am roots, with the majority displaying higher abundances in cultivated roots. These metabolic differences are likely influenced by environmental and geographical factors associated with the growing regions. Notably, the collection sites for natural and cultivated plants are separated by over 1300 km, with Zavkhan province representing a semi-arid and desert-like environment, whereas Khentii province features a forest-steppe ecosystem. Such contrasting ecological conditions, including differences in climate, vegetation type, and soil characteristics, are anticipated to contribute to the observed metabolic divergence.

In support, a previous study reported that both overall metabolite profiles and accumulation of major bioactive saponins in *Astragalus* roots are influenced by the soil quality, pH, microbial communities, and water availability (Li et al. 2022a). It is, therefore, essential to extend future comparative studies by focusing on bioactive metabolites, particularly saponins and flavonoids, using a liquid chromatography-based targeted metabolomics approach. This will provide a more accurate evaluation of phytochemical quality between natural and cultivated *A. membranaceus* roots. Yet, the current untargeted metabolite profiling has identified potential marker metabolites that can distinguish the n-Am and c-Am roots.

### n-Am roots: metabolic sources towards stress tolerance and energy balance

In both natural and cultivated *A. membranaceus* roots, metabolic pathways linked to the enriched and differentially accumulated metabolites exhibited overlaps. This could indicate that the specifically accumulated metabolites, either in n-Am or c-Am, were to support the major enriched pathways in their roots.

In n-Am roots, enriched pathways were functionally associated with three major roles: (i) Stress tolerance, involving the synthesis of cell wall polymers and stress responses (galactose metabolism and inositol phosphate metabolism), defense against pathogens (vitamin B6 metabolism and linoleic acid metabolism), mitigation of oxidative damage (ascorbate and aldarate metabolism), and acclimation to drought or salinity stress (arginine and proline metabolism); (ii) Biosynthesis of secondary metabolites, supported by galactose metabolism, fructose and mannose metabolism, biosynthesis of unsaturated fatty acids, ascorbate and aldarate metabolism, and arginine and proline biosynthesis; (iii) Energy production, growth and survival, facilitated by nitrogen assimilation (alanine, aspartate, and glutamate metabolism) and energy storage and mobilization (starch and sucrose metabolism). Collectively, metabolite accumulation and biosynthesis in n-Am roots are strongly associated with enhanced adaptive responses to abiotic and biotic stresses, similar to the main features observed in natural populations.

### c-Am roots: metabolic regulation towards central carbon metabolism and biomass production

In contrast to n-Am roots, the metabolic pathways in c-Am roots were primarily coordinated around primary metabolism and growth, with secondary metabolism playing a relatively smaller role. Functionally, the enriched pathways in c-Am roots contributed to: (i) Energy and carbon metabolism, driven by the citrate cycle, pyruvate metabolism, pentose phosphate pathway, and glyoxylate and dicarboxylate metabolism, supporting energy production, protein synthesis, and carbohydrate generation; (ii) Biomass accumulation, supported by nitrogen assimilation and amino acid metabolism via glycine, serine, and threonine metabolism; (iii) Maintenance of redox homeostasis and stress response, facilitated by redox balance and mild stress signaling, benefiting from ascorbate, lipoic acid, linoleic acid, and butanoate metabolism. These results suggest that cultivated *A. membranaceus* roots maintain a more balanced metabolic state, likely due to relatively stable agricultural environments with reduced environmental stress.

The observed differences in enriched metabolic pathways between natural and cultivated *A. membranaceus* roots may result from acclimatory metabolic reprogramming in response to distinct environmental conditions. Furthermore, variations in soil environments can directly influence the accumulation of essential macro- and micronutrients in plant tissues (Wang et al. 2022). Such changes in nutrient availability can, in turn, affect the biosynthesis and accumulation of bioactive metabolites in medicinal plants. For example, reduced nitrogen supply has been reported to increase the total phenolic content in the medicinal plant *Artemisia argyi* (Wang et al. 2023). Therefore, analyzing the mineral nutrient compositions in natural and cultivated *A. membranaceus* plants is important for better understanding their impact on metabolite profiles and for evaluating the nutritional quality of the roots.

## Conclusions

In conclusion, our comparative metabolomic analysis showed that, while natural and cultivated *A. membranaceus* roots contained similar levels of polysaccharide and monosaccharides, their metabolic profiles and accumulation patterns were distinct. A diverse array of metabolites, including sugars, sugar acids, amino acids, amines, fatty acids, and secondary metabolites, accumulated differently in natural versus cultivated roots, reflecting differences in metabolic regulation, environmental adaptation, and growth conditions. These findings offer valuable insights into the metabolic diversity of *A. membranaceus* and provide an important reference for evaluating, authenticating, and ensuring quality control of both natural and cultivated materials, supporting their effective medicinal and pharmacological use.

## Supplementary Information

Below is the link to the electronic supplementary material.

Supplementary Material 1: Supplementary Table (1) List of differentially accumulated metabolites in n-Am and c-Am roots. Supplementary Table (2) Metabolic pathways associated with enriched metabolites in n-Am and c-Am roots. Supplementary Table (3) Metabolic pathways associated with differentially accumulated metabolites in n-Am and c-Am roots.

## Data Availability

The datasets used and/or analyzed during the current study are available from the corresponding author on reasonable request.
